# Faith Cures

**Published:** 1885-12

**Authors:** 


### Editorial.

### FAITH CURES.

From time to time there is published in the secular papers an account of cases in which remarkable cures are claimed to have been suddenly wrought by the exercise of religious faith, or in answer to fervent prayer.

These accounts attract popular attention, provoke comment and challenge professional notice.

It is easy to deny the truth of them, or attribute them to the ignorance, deceipt or superstition of the witnesses; it is difficult to examine each case so as to verify the alleged facts; circumstances which would carry conviction to the laity would often be rejected by the medical observer, or interpreted by well-known physiological laws. The conclusion of these different classes would not likely be, and are not generally in accord.

The data for an exhaustive examination of the subject are not now attainable, but without denying that it is possible, in some cases, that "the prayer of faith can heal the sick," it must be admitted that the burden of proof rests upon those who assert it. In every case it must be proven that disease existed; that it was cured, and by that instrumentality. In few, if in any cases, has such proof been submitted.

In nearly all the examples of faith-cure, the evidences of disease have been wholly subjective, the feelings, sensations, emotions of which the subject alone can be conscious. They belong generally to the class of so-called nervous disorders, many of which can be feigned, simulated or imagined, and demand the very highest diagnostic skill to determine their existence. In no case, it is believed, have diseases, the existence of which is disclosed by objective signs, been made the subject of these experiments. Inflammation, small-pox, diphtheria, scarlet-fever, morbid growths, or other diseases recognizable by external signs, are not, as a rule, trusted to the power of faith alone.

As the existence of disease rests alone upon the testimony of the sufferer, the fact of the cure stands upon the same basis—a most unstable foundation in both. As it is easy to feign sensations, or describe imaginary sufferings, it is equally so to state that they have ceased, or exist no longer. To constitute evidence, both the fact of the disease and of the cure must be clearly established.

If both these points are proven or admitted, a more difficult task remains to show that the cure was produced by the alleged instrumentality.

In the practice of medicine there are few things harder than to form a just opinion of the effect of material agents in the cure of diseases. The task is greater when the agencies employed are immaterial and subject to no measure of force or quantity.

Spontaneous recoveries, in many diseases, are not uncommon, and especially in that class believed to be most amenable to the faith cures. Physicians are so often misled in attributing these to the effect of the drugs they administer, that they will be cautious in admitting the value of agencies of which they know nothing.

It is not surprising that these alleged faith-cures obtain credence with the populace. The world has been, from early historic times, filled with what are now called superstitions of like character and pretensions. Charms, amulets, incantations, abracadabra, and other like devices were long used for the prevention or cure of diseases, and when the authority of the church placed them under its band, other and simpler usages supplied their place, and these are still practiced amongst mankind; hundreds of these foolish superstitions, firmly trusted to, by large numbers of people. The personal testimony in favor of their curative and prophylactic powers is immense.

The royal touch for the cure of scrofula is no longer practiced; the healing well at Cripple-Gate is no longer thronged by the halt. But multitudes still resort to popular shrines to be healed; bones of saints inspire credulity, and thousands of ridiculous practices are employed by the people in all classes of socicty with confidence as strong, and boasted results as wonderful, and far outnumbering those claimed for "the cure by faith."

It cannot be denied that recoveries follow the employment of some or any of these ridiculous expedients, but until it is proven that they are actually produced by them, it is unnecessary to attempt to give the rationale of their action.

The power of the mind over bodily action, and especially of the imagination, in diseases whose manifestation consists in perverted nervous action, is well known, and often relied upon by the skillful physician as a means of medication, and it would be interesting to inquire how many of the alleged cures by "faith," or otherwise become rational and intelligible in this view of the subject, but until the essential facts of each case are established, it is impossible to apply to them the principles of physiological and psychological sciences.

### ITEMS.

Dr. OSCAR R. FORD, formerly of Rome, Ga., died recently in Kansas.

DR. HOWARD J. WILLIAMS, of Macon, is the happy father of a little daughter.

WE had a pleasant call from Dr. A. W. Wilder, of Prairie View, Ark., some days ago.

DR. J. B. PENDERGRASS, of Jefferson, Ga., was in Atlanta a few days ago.

THERE is a larger number of students in the Atlanta Medical College than for several years past.

THE *Century*, for November, is on our table, and, like every issue, is full of valuable matter. It is truly the leading literary magazine of America.

THE venerable Dr. John M. Johnson, of this city, who has been confined to his room for a long time with asthma, has so far recovered as to be able to ride out.

THE municipal authorities of this city have passed an ordinance prohibiting hotel and boarding-house proprietors from turning off the gas at the meter during the night.

CORRECTION.—In Dr. Nunn's letter in the October number of THE JOURNAL, he failed to credit Panola, DeKalb county, with one member of the Association.

DR. EUGENE FOSTER, of Augusta, contributes a valuable article to the first volume of The Reference Hand-book of the Medical Sciences, recently issued from the press of Wm. Wood & Co., New York.

DR. A. H. SHI, a member of the Medical Association of Georgia since 1860, is now living in Hawthorne, Fla. His many friends in Georgia will be pleased to learn that he is doing well in his new home.

WE had a pleasant call some time since from Dr. J. W. Clements, of Subligna, Ga. He says he is delighted with the action of the Association in adopting THE JOURNAL as the official organ of the Association.

THOSE of our readers who desire a first-class daily or weekly newspaper are referred to the card of the Savannah *News* among the new advertisements in this issue. We will club THE JOURNAL with either at low rates.

W. H. MARRETT, representing the popular publishers, Lea Bros. & Co., of Philadelphia, is canvassing the physicians in Georgia in the interest of that valuable work, System of Medicine, by Pepper.

WE are indebted to Dr. A. G. Hobbs, of this city, member of the American Rhinological Association, for a synopsis of the proceedings of the third annual meeting of the Association, held in Lexington, Ky., in October last.

DEATH OF MRS. DR. HOWELL.—This excellent lady, wife of Dr. D. H. Howell, of this city, died November 13th of peritonitis, after a few days' illness. We extend to the bereaved husband our warmest sympathies.

THE thirteenth annual meeting of the American Public Health Association will convene at Washington, D. C., Tuesday, December 8th, at 10 o'clock a. m. and continue four days. The meeting will be held in Willard's Hotel Hall on Pennsylvania Avenue.

DR. JOSEPH A. EVE, of Augusta, has resigned the professorship of obstetrics in the medical department of the University of Georgia, which he has filled for over fifty years, and Dr. W. H. Doughty, Jr., has been elected to fill the chair.

INOCULATION against yellow-fever, as practiced by Dr. Domingro Freire, at Rio de Janeiro, under the auspices of the Brazilian government, is fully explained in his communication to Dr. J. McF. Gaston, forwarded to Dr. Joseph Holt, President of the Louisiana Board of Health, who intends to bring this important subject before the approaching meeting of the American Health Association in Washington.

PROFESSOR W. F. WESTMORELAND presented at his clinic, at the Atlanta Medical College recently, a case of dislocation, of both bones of the forearm at the elbow, of three months' standing. The dislocation was reduced, and the patient experienced very little inconvenience afterwards.

LEONARD'S PHYSICIAN'S POCKET DAY-BOOK, FOR 1886, published by the *Illustrated Medical Journal* Company, Detroit, has been received. It is now in its ninth year of publication. It is good for *thirteen* months, from the first of any month that it may be begun, and accommodates daily charges for fifty patients, besides having cash department, and complete obstetric records.

WILLIAM BENJAMIN CARPENTER, M. D., LL. D., F. R. S., F. L. S., F. G. S., the eminent English physician, physiologist, and writer on scientific subjects, died in London, November 9th, from the effects of terrible burns caused by the upsetting of a lamp while he was taking a vapor bath for rheumatism.

AT a recent meeting of the Macon Medical Society, the following officers were elected : President, Dr. K. P. Moore ; Vice-President, Dr. E. G. Ferguson; Secretary and Treasurer, Dr. R. O. Cotter; Corresponding Secretary, Dr. H. McHatton; Librarian, Dr. C. H. Hall; Reporter, Dr. H. McHatton. The Society was never in a more prosperous condition than now.

THE MEDICAL NEWS VISITING LIST.—A complete pocket-book of useful memoranda for physicians and surgeons, with blanks suitable for keeping the professional and business records of a practice aggregating thirty patients per day. Wallet form, handsome red seal binding, tucks, pocket, pencil and rubber, $1.00. With patent thumb-letter index for rapid use, 25 cents additional. It contains desirable information and features which are to be found in no other visiting list we know. The patent thumb-letter index renders it especially convenient.

TOO MUCH FOR HIM.—The professor of anatomy, in one of the medical colleges in this city, recently asked a student what bones the sphenoid bone articulated with? He answered, "with all the bones of the body, except the coccyx, and in some rare instance with that."

LINDSAY & BLAKISTON'S Physician's Visiting List, for 1886, is on our table. This is the thirty-fifth year of its publication. Besides other valuable matter it contains a calendar, list of poisons and antidotes, dose tables rewritten in accordance with the sixth revision of U. S. Pharmacopœia, Marshall Hall's ready method in asphyxia, lists of new remedies, Sylvester's method for producing artificial respiration, with illustrations, diagram for diagnosing diseases of heart, lungs, etc., etc.

MEDICAL ENTERPRISE IN THE SOUTH.—Among many other indications of returning prosperity to the South is to be noticed the establishment of private infirmaries under the care of Southern physicians and surgeons. In Georgia, Dr. V. H. Taliaferro has in successful operation a private infirmary for the treatment of diseases of women. Dr. Taliaferro must find this a congenial field for his energy and talents, as his experience has been large and his success great in this department of medicine. More recently a surgical infirmary for males and females has been established in Atlanta by the well-known surgeon and writer, Dr. J. McF. Gaston, than whom no one stands higher in all sections of the country. In this connection reference may also be made to the deservedly renowned establishment of Dr. Battey, a description of which recently appeared in THE ATLANTA MEDICAL AND SURGICAL JOURNAL.—*Gaillard's Medical Journal, New York.*

MATERIA MEDICA COLLECTION FOR PHARMACAL AND MEDICAL STUDENTS.—The ever enterprising firm, Messrs. Parke, Davis & Co., of Detroit, Mich., offer a handsomely arranged case containing 288 specimens of *all* the crude drugs of vegetable origin recognized in the United States Pharmacopœia, and many not so recognized that are in common use, for the very moderate price of $10. This collection furnishes the indispensable supplement to such text-books as Maisch's *Manual*, Sayre's *Conspectus of Organic Materia Medica*, etc. Each of the 288 specimens is put up in a little neatly turned wooden box with a label bearing a number which refers to an index or key accompanying the case. Such a collection would seem an essential in the armamentarium of botanical and pharmacological students especially, and a great help to professors and teachers in colleges and schools. As each specimen selected is of unquestionable authority, this collection would also prove of great value to druggists as a means of testing their purchases of crude vegetable drugs by an authentic standard. Letters of inquiry and orders for the "Collection" should be sent *at once* to Messrs. Parke, Davis & Co., in order that they may know how many cases to have manufactured.

METEOROLOGICAL SUMMARY FOR OCTOBER, 1885, STATION, ATLANTA, GA.

| | |
|---|---|
| Highest Temperature, October 16th, . . . . . . . . | 73.9 |
| Lowest Temperature, October 22d, . . . . . . . . | 37.0 |
| Mean . . . . . . . . . . . . . . . . . . . . . | 56.5 |
| Greatest Daily Range of Temperature, October 16th . . | 26.7 |
| Least Daily Range of Temperature, October 11 . . . . | 4.8 |
| Mean Daily Range of Temperature, . . . . . . . . . | 15 9 |
| Mean Daily Dew-point, . . . . . . . . . . . . . . | 48.2 |
| Mean Daily Relative Humidity, . . . . . . . . . . | 76.3 |
| Prevailing Direction of Wind, . . . . . . . . . . | N. W. |
| Total Movement of Wind, . . . . . . . . . . . | 7,251 Miles. |
| Highest Velocity of Wind and Direction, . . . | 27 Miles, W. |
| Number of Foggy Days, . . . . . . . . . . . . . | None. |
| Number of Clear Days, . . . . . . . . . . . . . . | 16 |
| Number of Fair Days, . . . . . . . . . . . . . . | 9 |
| Number of Cloudy Days, . . . . . . . . . . . . . | 6 |
| Number of Days on which Rain or Snow fell, . . . . . | 8 |

